# Exploring the perspectives of older adults who are pre-frail and frail to identify interventions to reduce sedentary behaviour and improve mobility: a thematic content analysis

**DOI:** 10.1186/s12889-024-19051-2

**Published:** 2024-06-13

**Authors:** Isabel B. Rodrigues, Priscilla Ching, Mayank Kalra, Rong Zheng, Alexander Rabinovich, Alexandra Papaioannou, Carolyn Leckie, Dylan Kobsar, Qiyin Fang, Steven Bray, Jonathan D. Adachi

**Affiliations:** 1 Rady Faculty of Health Sciences, Department of Community Health Sciences, University of Manitoba, Winnipeg, MB Canada; 2 Faculty of Health Sciences, Department of Medicine, McMaster University, Hamilton, ON Canada; 3 Osteoporosis Canada, Patient-Oriented Research (POR), Toronto, ON Canada; 4 Faculty of Health, Department of Kinesiology and Health Sciences, University of Waterloo, Waterloo, ON Canada; 5 Faculty of Engineering, Department of Computing and Software, McMaster University, Hamilton, ON Canada; 6 Department of Surgery, Division of Orthopaedic Surgery, McMaster University, Hamilton, ON Canada; 7 Department of Health Research Methods, Evidence and Impact, McMaster University, Hamilton, ON Canada; 8 Faculty of Science, Department of Kinesiology, McMaster University, Hamilton, ON Canada; 9 Faculty of Engineering, Department of Engineering Physics, McMaster University, Hamilton, ON Canada

**Keywords:** Older adult, Frailty, Sedentary behaviour, Mobility, Qualitative description

## Abstract

Older adults who are frail are one of the most sedentary and the least physically active age groups. Prolonged sedentary time is associated with increased risk of negative health outcomes. To help design effective and sustainable content and optimize the uptake of sedentary behaviour interventions, an in-depth understanding of older adults’ perceptions of sedentary behaviour is needed; however, most qualitative studies have been conducted in healthy older adults. The aim of this study was to explore perspectives of sedentary behaviour within the context of older adults who are pre-frail and frail after the winter and spring. We included participants if they: (1) spoke English or attended with a translator or caregiver, (2) were ≥ 60 years, and (3) were frail on the Morley Frail Scale. We utilized a qualitative description methodology including a semi-structured in-depth interview and thematic content analysis. Concepts from the COM-B (Capability Opportunity Motivation–Behaviour) model were used to guide the semi-structured interviews and analysis. To ensure credibility of the data, we used an audit trail and analyst triangulation. We recruited 21 older adults (72 ± 7.3 years, 13 females, 13 frail) from southwestern Ontario, Canada. Two individuals were lost to follow-up due to medical mistrust and worse health. We transcribed 39 audio recordings. We identified three salient themes: (1) older adults rationalize their sedentary behaviours through cognitive dissonance (reflective motivation), (2) urban cities in southwestern Ontario may not be “age-friendly” (physical opportunity), and (3) exercise is something people “have to do”, but hobbies are for enjoyment despite medical conditions (psychological capability). Perspectives of sedentary behaviour were different in the winter versus spring, with participants perceiving themselves to be less active in winter. Incorporating dissonance-based interventions as part of an educational program could be used to target the reflective motivation and psychological capability components. Future research should consider interdisciplinary collaborations with environmental gerontology to develop age-friendly communities that promote meaningful mobility to target physical opportunity.

**Supplementary Information:**

The online version contains supplementary material available at 10.1186/s12889-024-19051-2.

## Background

Time spent engaging in sedentary behaviours are an important determinant of health [1]. There is moderate to high quality evidence that prolonged sedentary time is a risk factor for developing type II diabetes, obesity, heart disease, cancer, and premature mortality including all-cause, cardiovascular disease, and cancer mortality [2–5]. A systematic review of 22 studies from eleven regions (ten countries and the European Union) found that older adults accumulate an average of 9.4 h/day of sedentary time [6]. In older adults, greater levels of sedentary time have negative consequences on physical (e.g., functional impairment), psychological (e.g., cognition), and social (e.g., sense of belonging, loneliness) functioning [7]. We recently performed a systematic review comparing the sedentary behaviour and physical activity guidelines from around the world [8]. We found several sedentary behaviour guidelines recommend older adults limit sedentary time by replacing it with physical activity of any intensity [8]. While the benefits of physical activity are evident, there is some research in older adults that the time spent in sedentary behaviours are independent of the amount of time spent being physically active [9, 10]. There is little debate that prolonged sedentary time is associated with poor disease outcomes; however, patterns of sedentary time may differ within the same total amount of time (e.g., continuous sedentary behaviours without a break versus intermittent sedentary behaviours). There is a dearth of information about specific patterns of prolonged sedentary time and the effect of such patterns on one’s health [11]. More research is needed to understand how patterns of sedentary time may affect health especially in older adults.

Older adults who are frail are one of the most sedentary and the least physically active age groups [9]. Most of the time spent being sedentary is on leisurely pursuits within the home, and often in social isolation [12]. To date, only a few interventions have been developed targeting older adults’ sedentary behaviour [13]. Previous studies focused on reducing total sedentary time, while other studies aimed to increase physical activity levels with the assumption that sedentary time will be reallocated to physical activity [13]. A 2021 Cochrane review synthesized the results from seven studies (six randomized controlled trials and one cluster-randomized controlled trial) that targeted interventions to reduce total sedentary time among community dwelling older adults; the interventions focused on strategies like counselling, goal setting, and information sessions to reduce sedentary time and behaviour [14]. The authors of the Cochrane review concluded that it is not clear what interventions are effective at reducing total sedentary time in older adults. In addition, the effectiveness of such interventions on physical functioning are unclear and there are no studies on the effects of sedentary behaviour interventions on adverse events or mental health [14]. The Cochrane review also found there were no interventions that targeted individual behaviours or environmental and organization level policies that could inadvertently reduce sedentary time [14].

Older adults who are frail are potentially the population that might benefit the most from a reduction in sedentary time as they are the most sedentary group and have the highest chronic disease burden [15]. To inform effective sedentary behaviour interventions, an in-depth understanding of older adults’ perceptions of sedentary behaviour is needed. A 2020 systematic review of 15 studies pooled older adults’ perceptions of sedentary behaviour [16]. Ten studies involved healthy community-dwelling older adults and the other five studies were in older adults who had experienced a cardiac event or surgery, were obese, or had cancer [16]. But the perspectives of individuals who are pre-frail or frail may be different from those who are healthy. It is also important to understand older adults’ perspectives on which specific sedentary behaviours should be targeted, and when and where to intervene [14, 15]. Lastly, sedentary behaviours may differ by the season, which could change perspectives and has not been considered in previous studies. To develop sedentary behaviour interventions, the Knowledge-to-Action cycle [17] suggests adapting knowledge to the context as one of the first steps. Each section of the cycle is operationalized by a model, theory, or framework. For the purposes of this study, we used the Capability, Opportunity, and Motivation govern Behaviour (COM-B) model to operationalize the adapting knowledge to the context section of the cycle [18]. The COM-B model was chosen as it identifies the three major prerequisite factors involved in any volitional behaviour that describe why a behaviour occurs and, in turn, what factors may be targeted to facilitate behaviour change [18]. The aim of this study was to explore perspectives of sedentary behaviour in winter and spring and within the context of older adults who are pre-frail and frail.

## Materials and methods

This study is part of a larger research project, the MAPS-B (Mapping Sedentary Behaviour) study, which aims to understand the feasibility of measuring the context of sedentary behaviour among older adults who are pre-frail and frail [19]. To map the context of sedentary behaviour we used objective (wearable sensor and indoor positioning system), and self-report (daily diary) measures [19]. We used a valid thigh-worn sensor, activPAL4™, to distinguish between sedentary and standing postures [20]. We used a custom-designed indoor positioning system to obtain room level positioning information. The system was designed and validated to be used by older adults in their own homes without the need for a floor plan and only minimal initial setup and calibration; the system can also be used in homes with multiple stories with multiple residents [21]. Participants were equipped with the wearable sensor (activPAL4™) and an indoor positioning system, and completed a 24-hour diary of activities over three days (one weekend and two weekdays) in the winter (February 1, 2023 to March 21st, 2023) and spring (April 10th, 2023 to May 27th, 2023) [19]. The three measures were linked using date and time (an example of linking the data: sitting-living room-watching TV-alone weekend, Winter 3:30 pm to 5:15 pm). We registered the study on clinicaltrials.gov (NCT05661058) on December 22nd, 2022.

### Study design

We utilized a qualitative description methodology including a semi-structured in-depth interview and thematic content analysis [22]. Components from the COM-B model were used to guide the semi-structured interviews and analysis [18, 23]. The COM-B model is the starting point used by the Behaviour Change Wheel for understanding behaviour in the context in which it occurs [18]. Capability refers to the physical and psychological strength, knowledge, skills, and stamina needed to perform the behaviour [23]. Opportunity refers to the physical and social environmental factors (e.g., physically and socially accessible, affordable) to allow the behaviour to occur [23]. Motivation refers to the conscious and unconscious cognitive processes that direct and inspire behaviour [23]. We followed the COREQ 2007 guidelines for reporting of qualitative studies (Additional file 1) [24].

### Participants and settings

We received ethics approval from the Hamilton Integrated Research Ethics Board. All participants completed informed consent before enrollment. We included participants if they: (1) spoke English or attended with a translator or caregiver; (2) were ≥ 60 years and older; and (3) had a Morley Frail Scale score ≥ 3 (a score of 0 is robust, 1 or 2, pre-frail, and 3 to 5, frail) [25]. We excluded individuals who: (1) used a wheelchair for at least 55% of the awake day due to medical conditions; (2) were not independently mobile (i.e., require assistance from another individual to ambulate); and (3) had travel plans or other commitments that required missing > 30% of the rollout period. We sought to enroll both men and women as we anticipated gender may influence sedentary behaviour through socially constructed norms and roles and can be affected by differential access to resources, opportunities, and power. To ensure diversity in our recruitment process we partnered with CityHousing Hamilton Corporation, an organization that provides subsidized housing to low-income older adults many of whom are of visible minorities, immigrants, and have visible disabilities (i.e., use a walker or cane) [26]. We also recruited participants from physicians’ offices (family care and specialists), the local newspaper, and a local radio station from large metropolitan areas including Hamilton, Burlington, and Toronto. All cities are in southwestern Ontario, Canada. We also posted advertisements on social media using Facebook and Twitter. We recruited participants between January to February 2023.

### Positionality and intersectionality

In terms of positionality, the principal investigator (IBR) is a postdoctoral fellow at a major university in southwestern Ontario, Canada. IBR grew up in an affluent part of Toronto, Ontario where she attended a private Catholic school and participated in various athletic sports including the varsity team during her undergraduate. She is a second-generation immigrant whose parents were born in Africa and Asia. She identifies as a visible minority and is bilingual. IBR is an independent, early career researcher with experience in mixed-methods research including qualitative description, interpretive description, thematic content analysis, and framework analysis. She is a post-positivist whose ontological view lies between critical realism and bounded relativism, and her epistemology is constructionism. In terms of the positionality of the research team, we included a diverse group including researchers, healthcare professionals, patient partners, and trainees. Several members of the team identified from an underrepresented population (e.g., ethnic minority) whose first language was not English.

### Data collection

The principal investigator conducted semi-structured one-on-one interviews with participants after the winter (end of March and beginning of April) and spring (end of May and beginning of June) period (see Additional file 2 for interview guide). Before the start of the study, IBR spent several hours getting to know each participant to understand who they were, the participant’s goal for participating in the study, and to allow participants to ask questions to learn about IBR’s goals. Participants were provided with a visual feedback letter at least one week prior to the interview to give them enough time to reflect on the findings; the visual feedback letters were reviewed by three patient partners on our team to improve layout and clarity of the results (see Additional file 3 for sample visual feedback letter). All interviews were conducted over the phone. We were especially interested in exploring the types of sedentary behaviours participants were willing to modify and in identifying activities participants enjoy during warm and cold seasons. We mainly focused on sitting behaviours during awake hours as lying was associated with sleeping or napping for rest and may not need to be modified by an intervention. We used the COM-B model as an initial organizing framework to generate interview questions. In addition, we utilized questions from another qualitative study on office workers’ perspectives on sedentary behaviour [27]. Our patient partners reviewed and piloted the interview guide. IBR started the interview by reviewing the participant’s visual feedback letter including how much time, on average, the individual spent laying, sitting, standing, and walking over two weekdays and one weekend and the types of activities they engaged in while sitting. IBR then asked participants if they were surprised with their results (prompt: if “yes” or “no” why or why not it was surprising), their initial thoughts about the term sedentary behaviour. Specifically, the subsequent interview questions were then structured to probe elements of the COM-B model. Participants were asked to consider what makes sedentary behaviour “good” or “bad” (psychological capability), and what it means to be sedentary (physical capability). To capture physical and social opportunity, we asked participants if they had access to resources either at home or in the community that allowed them to be both sedentary and active and if there were social factors (e.g., support from family or friends, being part of a group) that aided their engagement in activities they enjoy. Lastly, we asked participants about what programs they would like to see in the community that would allow them to be more active and socially engaged (reflective and automatic motivation). Field notes were taken during the interview to help identify prompts. All interviews were audiotaped and transcribed verbatim. Demographic characteristics were collected using PROGRESS (Place of residence, Race/ethnicity, Occupation, Gender and sex, Religion, Education, Socioeconomic status, and Social capital) [28]. We also assess frailty scores and probable sarcopenia using the Fit-Frailty Assessment & Management Application (pre-frail scores 0.18 to 0.24 and frail > 0.24) [29].

### Data extraction and analysis

We analysed all interviews using thematic content analysis to generate a narrative description to cover key regularities and patterns evident in the data [22]. We selected thematic content analysis as it is a pragmatic approach that utilized an ‘etic’ interpretation [22]. Thematic content analysis involves four steps including (1) familiarize self with data, (2) identify themes, (3) code the data, and (4) organize codes to develop themes [30]. As described by Anderson 2007, IBR listened to each interview and read each transcript to gain a feel for the data including how participants talk about issues, recurrent topics, and broad typologies of experiences, events, and views [30]. The process began with predetermined codes and categories from the COM-B model. Coding was subsequently modified using an inductive approach to account for all the data. All transcripts were analyzed using latent content [31] and constant comparative techniques [22], which involved allowing themes to emerge from the data, refining the themes into conceptual categories, and exploring the interrelationships of these categories. All coding and theme development was performed by IBR. NVivo, version 14 (QSR International Pty Ltd, Doncaster, VIC, Australia) was used to manage the data and support our analysis.

### Data credibility

To ensure credibility of the data, we used an audit trail and analyst triangulation. An audit trail was created to describe the steps and document decisions that were made about the extraction process, as well as the identification of codes, categories, meaninful clusters, and key topics identified in the transcripts. Sufficient time was spent reviewing each transcript to identify recurrent patterns and key topics of the document. IBR spent extensive time reading and re-reading all transcripts, and took notes of emerging ideas for each transcript in a notebook after analyzing the fourth transcript (e.g., what is the main message?, how does the message relate to other transcripts?). To further enhance the credibility and dependability of the data, IBR engaged in reflexivity and documented her own biases, preferences and preconcpetions about the topic in a series of memos [32, 33]. We also employed analyst triangulation with a sample of four participants (two men and two women) who offered their perspectives about the preliminary and final findings [33]. IBR met with each of the four participants over six meetings, in person, to discuss the results and whether the key findings resonated or differed from their experiences. Lastly, one of the patient partners on our research team (PC) reviewed the final themes and provided feedback.

## Results

We enrolled 21 participants and interviewed 20 individuals after the winter period and 19 after the spring period. Two individuals were lost to follow-up; one individual dropped out after the initial study visit due to research mistrust in the wearable sensors and the second individual dropped out after the winter interview due to worsening health. Two individuals whose first language was not English attended each interview with a caregiver. We transcribed 39 audio recordings. The average length of the interview was 50 min with the shortest interview being 35 min and the longest interview being 1 h and 30 min. The mean age of participants was 72 ± 7.3 years and eleven participants self-reported they were living with osteoarthritis (Table 1). The mean temperature in the winter in southwestern Ontario was − 1.5 ± 4.74 ℃ (average high 1.4 ℃, average low − 4.5 ℃), while in the spring it was 10.4 ℃ ± 5.3 ℃ (average high 14.17 ℃, low 6.7 ℃). The average sedentary time spent during awake activities in winter was 10.1 h ± 2.9, while in the spring, 9.6 h ± 2.6. Individuals who were frail spent more time being sedentary on average during both seasons; however, their sedentary time was not significantly different from individuals who were pre-frail. Apart from differences in gait speed, semi-tandem, anxiety, and health-related quality of life scores, individuals who were frail did not vary from those who were pre-frail in terms of health-related outcomes. Three salient themes relating to the COM-B model constructs emerged from the interviews. Theme 1 relates to reflective motivation, which “involves planning (self-conscious intentions) and evaluations (beliefs about what is good and bad)” [23]. Specifically, participants shared generally negative evaluations of sedentary behaviour, but also experience internal conflict, or cognitive dissonance, as they rationalize the amount of time they spent sitting despite its health risks. Theme 2 captures physical opportunity, as the “opportunity afforded by the environment involving time, resources, locations, cues, and physical affordance” [23] with the data suggesting large urban cities in southwestern Ontario do not have age-friendly design characteristics. Theme 3 reflects psychological capability in reference to having “knowledge or psychological skills, strength, or stamina to engage in the necessary mental processes” [23], as participants shared an understanding that [23] exercise is something people must do, but hobbies are for enjoyment despite medical impairments.

**Table 1:** Demographic characteristics of participants reported using PROGRESS and other descriptive considerations (*n* = 21)

Mean age (SD), years	72 ± 7.3
Mean height (SD), cm	166.7 ± 11.2
Mean weight (SD), kg	77.2 ± 20.6
Female sex, n (%)	13 (62%)
Frail on Fit-Frailty app, n (%) Pre-frail on Fit-Frailty app, n (%)	13 (62%) 8 (38%)
Probable sarcopenia, n (%)	17 (81%)
Ethnicity, n (%)	
Caucasian South Asian East Asian	18 (85%) 2 (10%) 1 (5%)
Highest Level of Education, n (%)	
Grade school High school Higher education (college or university)	5 (24%) 6 (28%) 10 (48%)
Employment, n (%)	
Retired Medical leave Full-time (40 h/week)	19 (10%) 1 (32%) 1 (10%)
Annual income, 2023 CAD	
< 20,000 20,001 to 40,000 40,001 to 60,000 > 60,000	2 (10%) 7 (32%) 2 (10%) 10 (48%)
Place of Residence, n (%)	
In the community alone In the community with others Retirement home, alone	8 (38%) 12 (57%) 1 (5%)
Visit from friends and family, n (%)	
Daily Weekly Monthly	8 (38%) 8 (38%) 5 (24%)
Medical history, n (%)	
Cancer Cardiovascular Hearing impairment Joint disease Musculoskeletal condition Respiratory	6 (29%) 4 (19%) 8 (38%) 11 (52%) 9 (42%) 5 (24%)
Number of chronic conditions, n (%)	
1 to 2 3 to 4 ≥ 5	5 (24%) 9 (43%) 7 (33%)
Winter 2023	
Average Laying (hrs/day) Average Sitting (hrs/day) Average Standing (hrs/day) Average Walking (hrs/day) Average Step count (steps/day)	8.3 ± 1.6 10.1 ± 2.9 4.2 ± 2.1 1.3 ± 0.8 5699 ± 3557
Spring 2023	
Average Laying (hrs/day) Average Sitting (hrs/day) Average Standing (hrs/day) Average Walking (hrs/day) Average Step count (steps/day)	8.4 ± 1.1 9.6 ± 2.6 4.4 ± 2.4 1.5 ± 0.8 7170 ± 3924

### Theme 1 (Reflective motivation) - Rationalizing sitting: “Passive” sedentary behaviour should be distinguished from “active” or “purposeful” behaviours

Participants believed that sedentary behaviours are associated with negative health outcomes, and they used negative language to describe sedentary behaviours or individuals who are sedentary:

“*Someone who sits a lot on the couch and watches television or is just sitting down thinking about yesterday and things like that*” (frail male, 81 years, average sitting time in winter 7.9 h/day and spring 6.5 h/day).

Older adults used negative terms and idioms (e.g., lazy or couch potato) to describe passive sitting behaviours. Upon reflecting on their own sedentary behaviours, participants did not distinguish between sitting or not sitting. Rather they categorized their sedentary behaviours as either active with a purpose or not active with a purpose. Several participants believed their sitting behaviours should not be considered sedentary (or “bad”) because it was done with a purpose such as sitting for medical reasons or to accomplish a task (e.g., paying bills, painting) suggesting a lack of reflective motivation (beliefs about what is good or bad):

“*Like even though I’m at home, I’m busy or if I sit at my desk doing something… like I do art or I do sketching, that kind of stuff then. That’s about as much sitting as I do or if I’m eating a meal.”* (prefrail female, 75 years, winter: device malfunction, spring: 6.2 h/day).

When considering gender differences, men, in particular, felt that the amount of sitting in their visual feedback letter was unusual and some men questioned if the activPAL4™ monitor was accurate:

“*That doesn’t sound… that does not sound right! <*interviewer asks what does not sound right*>. The sitting part because <* retract wife’s name *> got her affliction… I’m the one that’s doing all the things that a house needs from laundry, putting it away, etc., so on and so forth so… that one there has got me bamboozled…. why it would be only that!*” (frail male, 74 years, winter: 11.4 h/day, spring 11.1 h/day).

Participants who identified as female also disagreed with the amount of sitting captured by the activPAL4™ device, but rather than suggest the device was inaccurate, they rationalized their sitting behaviours as purposeful:

“*Well see I don’t agree with the 11 hours. And the reason I say that is first of all, those 11 hours I’m really not just sitting….I’m one of those that while watching TV I’ll play spider or something like that on my iPad, or I’m checking my email and stuff like that. And I have the dog… so the dog… you’re up and down with the dog you know what I mean, getting him a treat or whatever like that. But no, I know I sit far too much but it’s at home…. and it’s… like I love to do things. I don’t like just sitting and doing nothing*” (frail female, 79 years, winter: 11.9 h/day, spring: 11.2 h/day).

Participants emphasized the importance of keeping ‘mentally active’ especially as a retiree. There was a clear distinction between active sedentary behaviour (e.g., watching a reputable documentary) versus passive sedentary behaviour (e.g., watching TV that does not mentally stimulate the mind). Participants rationalized their own sitting practices by distinguishing between passive versus active sedentary behaviours.

### Theme 2 (Physical opportunity) - Limited access to “age-friendly cities”: Adverse weather conditions coupled with inaccessible public spaces promotes sedentary behaviour and social isolation in winter

Although there were no significant differences in sitting time between winter (10.1 ± 2.1 h/day) and spring (9.6 ± 2.6 h/day), participants perceived they were more sedentary during the winter: “*in the minuses, I don’t function at all*” (frail male, 68 years, winter: 5.3 h/day, spring: 5.7 h/day). There was a strong perception that the warmer months were associated with being more physically active because of the time spent outdoors engaging in activities such as gardening, walking in the park, and visiting friends:

“*You know, sometimes it sort of all depends on weather and stuff, so I know that certainly during the late spring, summer and fall months I tend to spend a lot of time outdoors so I’m puttering around in my garden and that kind of thing than certainly… than I do in the winter months. I know that I spend probably a lot more time sitting over the colder months*” (frail female, 72 years, winter: 10.0 h/day, spring: 9.1 h/day).

During the winter months, older adults spend more time indoors and at home, while during the warmer months they spend time outdoors indicating seasonal differences contribute to physical opportunities (or lack thereof). There was a perception that having access to an outdoor space promotes movement and activity. Older adults were fearful of being active during the winter and used negative language to describe winter activities such as “dangerous” or “fear of falling”. Most older adults spent time indoors during the winter, which promoted a sense of loneliness and social isolation:

“*But winter you know, there’s not much we can do. To go out it’s really, really cold, it’s so windy. I can feel, especially when the days are damp and humidity, I can feel in my bones the humidity and I get really, really cold. I’m in pain, I’m kind of depressed I don’t feel good*” (frail female, 65 years, winter: 5.2 hours/day, spring: 4.1 hours/day).

After a snowfall, it is standard for urban cities in southwestern Ontario, Canada to clear bike and car lanes to ensure the safety of motorists; however, the residual snow is often piled in front of homes or on the sidewalk, making it a challenge for older adults to leave their homes or use the sidewalk. A major concern raised by several participants was the fear of falling or fracturing. During the interviews, participants described major urban cities in southwestern Ontario as not being “age-friendly” especially in the winter and colder months:

“*Well like I said in the wintertime, it was wonderful that they cleaned the streets, it was wonderful that they cleaned out the bike lanes, but they made a mess of everything else. One of the first snowfalls that we had, I spent the next morning shoveling out because I had some library books to go back, and I left my car on the street because I knew if I parked it back in my driveway, everything off the road would get dumped and I would never get my car out. But I walked to the library so it’s the <* retract name of library*>, so it is about a 2 km walk to the library and back. There was not one street, not one corner that I could cross that wasn’t… just had everything that was dumped by the plows into…. so you couldn’t walk across and my balance isn’t the best these days either, so at one point I started walking in the bike lanes because they were cleaned out*” (prefrail female, 72 years, winter: 13.2 h/day, spring: 12.6 h/day).

The absence of safe and accessible outdoor space, especially during winter, prevents participants from partaking in activities they enjoy (e.g., walking). Unfavourable weather conditions and inaccessible public spaces may be promoting inactivity and social isolation.

### Theme 3 (Psychological capability) - Interventions to target sedentary behaviour: Exercise is something *you must do*, while hobbies are for enjoyment regardless of medical history

Most participants unenthusiastically suggested exercise as a method to reduce sitting time during the winter and spring months. Participants described exercise as something “I should do” or “I know I have to do”. When prompted on the types of exercise programs participants would like to see in the community, several older adults admitted they would not follow through with a structured exercise program, which may indicate a lack of knowledge or psychological skills/stamina. Most participants only engage in a structured exercise program or sought physical therapy after a major health incident:

“*To be quite honest I would probably say yes and then I wouldn’t follow through. <*interviewer asks why they would not follow through*>. I don’t know. Maybe it’s because we’re lazy.* < interviewer asks what they mean by ‘lazy’>. *When I’m thinking when you ask about that is… that a long time ago my husband and I both were doing physical therapy, I had a shoulder problem… he was doing it to strengthen his hip and his balance for walking and we were both supposed to carry on once we finished going to therapy and neither one of us really did. Once it got better, we just quit so we should have kept it up. So that’s what I’m thinking is I think I might do it but I don’t know that I really would*” (frail female, 80 years, winter: 9.1 h/day, spring 9.4 h/day).

When asked about activities participants enjoy, they enthusiastically described partaking in light physical activities such as Nordic walking, dancing, lawn bowling, tai chi, yoga, gardening, swimming, visiting family and friends, and volunteering especially during the warmer months. Some older adults enjoyed activities with a fun but competitive component. Several participants were interested in joining a Nordic walking group with a social component during warmer months as a method to decrease their sedentary time. Participants used positive language to describe such activities as “hobbies” rather than use the terms exercise or physical activity. Some participants were willing to engage in hobbies despite pain from medical conditions.

“*Well, I held off because there was going to be a frost and planting the border is a big job. It took me three hours and it was painful because I had to sit because of my bad knees <* referring to osteoarthritis > *on the sidewalk and dig and then planting a plant. <*Interviewer asks why they garden despite the pain>. *I like the way it looks, that’s the trouble. It’s a lot of work and I like the way it looks*” (prefrail female, 71 years, winter: 16.8 h/day, spring: 13.1 h/day).

Participants had questions for our research team about the best types of activities to offset the negative health effects of sedentary behaviour. Some individuals considered continuous walking could be beneficial while others believed short bout of getting up (e.g., sit-to-stand) could offset the negative health effects of too much sitting. Older adults are enthusiastic about participating in activities they enjoy despite experiencing pain or having a chronic disease.

## Discussion

Sedentary behaviour has become an important topic within public health messaging. Using a qualitative description methodology guided by the COM-B model, we attempted to understand participants’ perspectives of their own sedentary behaviours and how those behaviours may differ in the winter and the spring. We found that many older adults who were pre-frail or frail understood the consequences of sedentary behaviour, but rationalized their behaviours as “purposeful” and “active”/“busy” (i.e., good behaviours). This theme may be linked to the reflective motivation component in the COM-B model, where participants believe their sedentary behaviour to be “good”, while the behaviours of others to be “bad” [23]. Although we found no significant differences in sitting time between winter and spring, participants perceived they were more sedentary during the colder months due to inaccessible public spaces. We anticipate there may be differences in sedentary behaviour across seasons; however, our study did not allow enough time between seasons to capture these differences. The lack of physical opportunity and environmental resources may be restricting older adults from engaging in light physical activities during the cooler months. Lastly, participants understood the importance of exercise but preferred to engage in lifestyle programs or hobbies despite having persistent pain or medical conditions. There was this belief (i.e., psychological capability) that structured fitness programs may not be the best method to reduce sedentary behaviour, despite the value and benefits of exercise.

Participants in our study associated the term “sedentary behaviour” with negative connotations and they clearly understood the health consequences of too much sitting; however, despite having high sedentary times themselves, they sought to rationalize their sedentary behaviour by distinguishing between ‘active’ versus ‘passive’ behaviours. Our findings bear similarity to other studies that aimed to understand perspectives of sedentary behaviour in healthy older adults. McGowan et al. 2020 reported that older adults in their study viewed sedentary behaviours as having negative connotations particularly when watching a lot of television [34]. Similarly, Palmer et al. 2020 found older adults moralize sitting by distinguishing different (active/‘busy’/worthwhile versus passive/‘not busy’) types of sitting [35]. The creation of “good” versus “bad” sedentary behaviours allows older adults to set themselves apart from other individuals whose sedentary behaviours may be considered deviant (e.g., lazy couch potato) [35]. This distinction between “good” and “bad” lends itself to a lack of reflective motivation regarding “beliefs about what is good and bad” [23]. Participants in our study may be experiencing cognitive dissonance as they do with other public health information such as smoking, diet, and medical screening [36]. Several of the men in our study questioned if the wearable sensor was accurate, while the women attempted to justify their sitting by describing it as purposeful and active sitting. Although some cognitively engaging sedentary behaviours (e.g., reading, socializing) may benefit health and time spent in more passive activities may be detrimental, prolonged time spent in sedentary behaviours are associated with poor health outcomes [2–5]. We still need more research about which specific patterns of prolonged sedentary behaviours result in negative health consequences [11]. Nevertheless, the barrier is not that there is a lack of knowledge that prolonged sedentary time has negative health outcomes, but rather we need interventions to target cognitive dissonance.

Intervention functions that are linked to reflective motivation include education, persuasion, incentivization, and coercion [23]. Educational programs alone aimed at disseminating knowledge on the negative health effects of sedentary behaviours may not be helpful to target cognitive dissonance as most older adults are aware that too much sitting can be harmful. Our study and the Palmer et al. study [35] found older adults understood the negative effects of sedentary behaviours but believed that their sitting was different. To target the cognitive dissonance that older adults experience, dissonance-based interventions as part of an educational intervention may be beneficial. Most dissonance-based interventions include a combination of verbal, written, and behavioural exercises to create dissonance to target the behaviour. One study utilized a dissonance-based intervention to target obesity and food intake [37]. The intervention utilized a persuasion intervention function consisting of six 1-hour group meetings with six to ten participants, during which participants were encouraged to support other individuals in adopting lifestyle changes [37]. The interventions included verbal, written, and behavioural exercises meant to promote healthy lifestyles and create awareness of dissonance lifestyle choices that led to excessive weight gain [37]. During each session, participants discussed the negative effects of overeating high-calorie foods, sedentary behaviours, and other lifestyle choices that could contribute to obesity [37]. Including dissonance-based exercises in educational interventions could be an important component to decreasing sedentary behaviours in older adults.

In our study, older adults highlighted the limited number of “age-friendly” communities in major urban areas in southwestern Ontario. The lack of physical opportunity especially during the cooler seasons may be preventing older adults from engaging in light physical activities that they enjoy. Intervention functions associated with physical opportunity include training, restriction, environmental restructuring, and enablement [23]. Discussions with older adults indicate that environmental restructuring may be the most ideal intervention to target sedentary behaviour during colder months. Current interventions for older adults narrowly focus on implementing technology in the design specifications but considering the concept of age-friendly cities as a form of environmental restructuring in the research design may be critical to improving mobility, social relationships, and quality of life. The importance of environmental gerontology in medical research can help accelerate knowledge translation interventions to increase mobility in older adults. The WHO (World Health Organization) first coined the term “age-friendly city” in 2005 when it launched the Global Age-Friendly Cities Project [38, 39]. Lui and colleagues published a comprehensive review of the international literature on age-friendly communities [40]. The review describes two facets that are necessary when considering age-friendly communities. One facet includes a continuum between the physical environment and social environment, while the other facet provides a spectrum on the type of governance (i.e., top-down and bottom-up) [40]. Several countries have developed and implemented age-friendly initiatives that utilize such facets. Models such as the National Association of Area Agencies on Aging (N4A) have focused on building the physical environment to address the needs of older adults like affordable housing, updated transportations services, accessible health and supportive services and community-based arts, culture and enrichment programs, and home modification programs [41]. Specifically, the transport services aim to remove the obstacle that the current “road design makes walking difficult” for older individuals [41]. Limited or inaccessible transportation was a common theme in our study, where older adults found it difficult to navigate large urban cities, especially after a major snowfall. The N4A suggests utilizing walkability audits to identify and prioritize pedestrian walkways [41]. Other age-friendly models include the United Kingdom Elaboration of Lifetime Neighbours Program, which focuses on the social environment to build accessible homes for older adults (i.e., the Housing and Older People Development Group) [42]. The housing group suggests building accessible gardens as an important component to encourage movement and reduce social isolation [42]. In our study, participants discussed the value of gardening despite having osteoarthritis that caused them ongoing discomfort and pain. When designing age-friendly cities and communities a bottom-up governance approach should be utilized as this type of governance considers the needs and values of older adults [40]. Facilitating older adult’s participation in the design of age-friendly communities empowers them and cultivates their capacity to enhance their neighbourhood/community in a meaningful way. Rather than have older adults “age in place”, we should consider helping “older adults age in a place” as older people should be able to successfully age wherever they choose to reside.

When we asked participants about what programs or activities would reduce their sitting time, most participants unenthusiastically suggested exercise. There was this dichotomous belief that sedentary behaviour could only be compensated with moderate or vigorous exercise such as a structured exercise program. Research suggests that interrupting one’s sedentary behaviour with light physical activity or postural changes throughout the day could also be beneficial [43–45], so the activities do not necessarily need to be moderate or vigorous. Light physical activity such as standing or walking slowly has been shown to increase metabolic rate in comparison to sedentary behaviours [43]. Participants’ limited knowledge regarding how to reduce sedentary behaviour may be an indicator of lacking psychological capability (i.e., the knowledge to reduce sedentary behaviour) [23]. Participants belief that sedentary behaviour can only be compensated with exercise may indicate a need to include an educational intervention on the benefits of engaging in light physical activity. During our interviews, participants listed several light physical activities/hobbies they would be willing to participate in. Educational programs should include information on lifestyle programs participants can engage in to reduce their sedentary time with an emphasis that the programs do not need to be moderate or vigorous.

The present study includes few limitations that provide opportunities for future research. The interpretive nature of our research constrains the generalizability of our results. While we provide rich description of the perspectives of older adults in southwestern Ontario, these findings do not necessarily apply to all regions of Ontario or even Canada such as rural areas or smaller cities where access to resources may differ. Although we attempted to recruit a diverse group of older adults, our study was limited to individuals of Caucasian descent. In addition, our study did not account for enough time between the winter and the spring collection period, so capturing sedentary behaviour in the summer and winter may reveal distinct patterns of sedentary behaviour that will be important to analyze for future research.

### Future research

Our study showed that the perspectives of individuals who are frail and pre-frail are no different from healthy, community-dwelling older adults in other studies. We anticipate that a specific intervention to reduce sedentary behaviour for individuals who are pre-frail or frail may not be necessary, but due to our small sample size more research on the frail population is needed. A major finding in our study indicates that there may be benefits to engaging in certain types of sedentary behaviours that should be considered simply beyond the fact that they are “sedentary”. Before we develop an intervention that targets sedentary behaviour, we should first identify which types of behaviours older adults value and prioritize as these behaviours may not need to be targeted in an intervention. Once we have identified which types of sedentary behaviours are valuable and important for daily living, researchers could then focus on targeting behaviours that older adults are willing to modify. Our study indicates that an intervention that includes an educational program with a cognitive-dissonance component on the benefits of breaking-up prolonged sedentary behaviour could be promising. The education program should focus on activities older adults enjoy such as light physical activities. Participants also mentioned the benefits of there being a social component that was mentally stimulating for enjoyment and motivation. If interventions are developed during cooler seasons, considering age-friendly programs that are accessible. Lastly, if sedentary behaviour interventions are implemented, seasonality should be considered such that an intervention should not be implemented in winter and outcomes assessed in spring or summer.

## Conclusion

Developing targeted interventions to reduce sedentary behaviour in older adults can have significant health benefits. The findings from this study provide novel insight on the perspectives of older adults who are pre-frail and frail. Our study has some unique components as we assessed the context of sedentary behaviour using objective and subjective measures and provided participants a visual feedback letter prior to their interviews after the winter and spring period. We identified three salient themes: (1) “passive” sedentary behaviour should be distinguished from “active” or “purposeful” behaviours (reflective motivation), (2) weather conditions coupled with inaccessible public spaces promotes sedentary behaviour (physical opportunity), and (3) exercise is something people “have to do”, but lifestyle programs are something people enjoy doing despite their medical condition (psychological capability). Future research should consider interdisciplinary collaborations with environmental gerontology to expand the concept of meaningful movement to help older adults age in a place. In addition, incorporating dissonance-based interventions may be an important component to disseminate educational material on the harmful effects of sedentary behaviour.

### Electronic supplementary material

Below is the link to the electronic supplementary material.

Additional file 1. COREQ 2007 guidelines for reporting of qualiative studies

Additional file 2. Semi-structured interview guide in winter and spring

Additional file 3. Sample visual feedback letter

## Data Availability

The data generated during the current study are not publicly available due to content that could compromise research participant privacy but are available from the corresponding author on reasonable request for research purposes only.

## Abbreviations

COM-B: Capability Opportunity Motivation–Behaviour
MAPS-B: MAPping Sedentary Behaviour
N4A: National Association of Area Agencies on Aging
PASS: Physical Activity, Sedentary Behaviour and Sleep
PROGRESS: Place of residence, Race/ethnicity, Occupation, Gender and sex, Religion, Education, Socioeconomic status, and Social capital
WHO: World Health Organization
