# Supplementary material for: Exploring the perspectives of older adults who are pre-frail and frail to identify interventions to reduce sedentary behaviour and improve mobility: a thematic content analysis
Source: BMC Public Health. 2024 Jun 13;24:1582. doi: 10.1186/s12889-024-19051-2 (PMC11170854; doi:10.1186/s12889-024-19051-2)
Supplement: Supplementary file 2 — Additional file 2. Semi-structured interview guide in winter and spring [file 12889_2024_19051_MOESM2_ESM.pdf]

Mapping how, where, and when sedentary behaviour occurs in older adults who are frail Study

Semi-Structured Interview Winter

1. I would like to start the conversation by reviewing the results of the study so far <review results from visual feedback letter>.
    a. What surprises you about the results presented here?
        i. Prompt: why/for what reason?
2. When you hear the term sedentary behaviour, what comes to mind?
    a. Prompt: what do you think of?
3. What does it mean to be sedentary?
4. What are the benefits of… [behaviour]?
    a. Prompt: What are the advantages of certain types of sedentary behaviours of you/for your health?
    b. Prompt: What (possible) disadvantages does… [behaviour] have for you/for your health?
5. What possible health consequences could there be for someone who does a lot of sitting/X behaviour?
    a. Prompt: Do you think they could be bad for someone's health? If "yes" or "no", why? Or do you consider it unimportant? Could you explain why you think this is the case and what effect this has on your body?
    b. How much movement does someone of your age need per week?
6. Are you happy with the amount of sitting you are doing?
    a. Prompt: Would you like to change something about your behaviour?
    b. Prompt: We are also interested in reasons why people do not want to change their behaviour.
7. Can you tell me why you want/don't want to change this? (Sometimes there are multiple reasons or the reason is not immediately obvious; you can take your time to think about this question).
    a. Why is this important/less important to you? Why do you value this?
8. How much time would you like to sit throughout the day?
    a. Prompt: Why that number?

9. What are things you experience that require you to sit for long periods of timer?
10. What are thing that you can do to break up your sitting time?
11. What do you need to achieve your goals regarding sedentary behaviour?
    a. How could your social environment help you to be less sedentary?
    b. How could your physical environment help you to be less sedentary?
    c. Would you like to receive more information about the health effects of sedentary behaviour? If so: what kind of information would you like to receive and how?
12. Which values/characteristics – directly or indirectly – influence the extent to which you are physically active or sedentary? Can you explain why?
13. What characterizes a ‘good life’ according to you?
14. We gave you asked you to wear two pieces of equipment and complete a diary. I am going to start of with the device that was attached to your leg.
    a. What did you like about the device on your leg?
    b. What did you dislike?
    c. We gave you a system that had a series of black boxes and watch – what did you like and dislike about the system?
    d. Did you use the electronic diary? What did you like or dislike?

End of the interview

1. Would you like to add anything we have not yet discussed but might be of relevance for this interview?
2. Do you have any further questions?

Thank you very much for your participation in this interview. Your answers are valuable and may be used to improve lifestyle programs that are in line with people’s values and needs.

Semi-Structured Interview Spring

1. Like the last interview I would like to start by reviewing your spring results and comparing them to the winter results < review results from spring and winter visual feedback letter >.
2. Did you expect your sitting time to be different in the winter versus the spring?
3. Did the results from this study surprise you?
    - i. Prompt: why/for what reason?
4. Now that the weather is warmer, what are some activities that you will be doing?
5. When the weather is cooler what activities do you enjoy doing?
6. What encouraged you to join the study?
7. Do you have any suggests on how we could involved you more in the study?
8. We gave you asked you to wear two pieces of equipment and complete a diary. I am going to start of with the device that was attached to your leg.
    - a. What did you like about the device on your leg?
    - b. What did you dislike?
    - c. We gave you a system that had a series of black boxes and watch – what did you like and dislike about the system?
    - d. Did you use the electronic diary? What did you like or dislike?

End of the interview

Would you like to add anything we have not yet discussed but might be of relevance for this interview? Do you have any further questions?

Thank you very much for your participation the study. It was a great pleasure to get to know you and to learn from you.

Thank you again. Have a nice day.
