# Supplementary material for: Exploring the perspectives of older adults who are pre-frail and frail to identify interventions to reduce sedentary behaviour and improve mobility: a thematic content analysis
Source: BMC Public Health. 2024 Jun 13;24:1582. doi: 10.1186/s12889-024-19051-2 (PMC11170854; doi:10.1186/s12889-024-19051-2)
Supplement: Supplementary file 3 — Additional file 3. Sample visual feedback letter [file 12889_2024_19051_MOESM3_ESM.pdf]

McMaster University HEALTH SCIENCES

Department of Medicine, Division of Geriatric Medicine
St. Peter's Hospital
158-88 Maplewood Avenue
Hamilton, ON L8M1W9

Dr. Isabel B. Rodrigues
(905) 521-2100 EXT 12464
rodrigibl@mcmaster.ca

Project Title: Mapping Sedentary Behaviour (MAPS-B) in Older Adults who are pre-frail and frail.

Lead Investigator: Dr. Isabel B. Rodrigues PhD MSc
Department of Medicine, McMaster University
Phone: 905-521-2100 EXT 12464
Email: rodrigib@mcmaster.ca

Funding Source: Hamilton Health Sciences New Investigator Fund

Dear <insert participant's name>

We would like to thank you for enrolling in our study. The MAPS-B study looked at the kinds of sedentary behaviour you do. Sedentary behaviours are things you do while awake that use little energy. Examples of sedentary behaviour may be eating breakfast at the kitchen table. Or watching your favourite TV program. Or knitting your granddaughter a scarf on the sofa. There is some research that says sitting for too long may be bad for our health. Our research team will use your result to make community lifestyle programs to reduce sedentary behaviours.

Below and on the next page, please find a summary of your results from the **winter period**:

Average 24-hour Winter Activity over 3 days

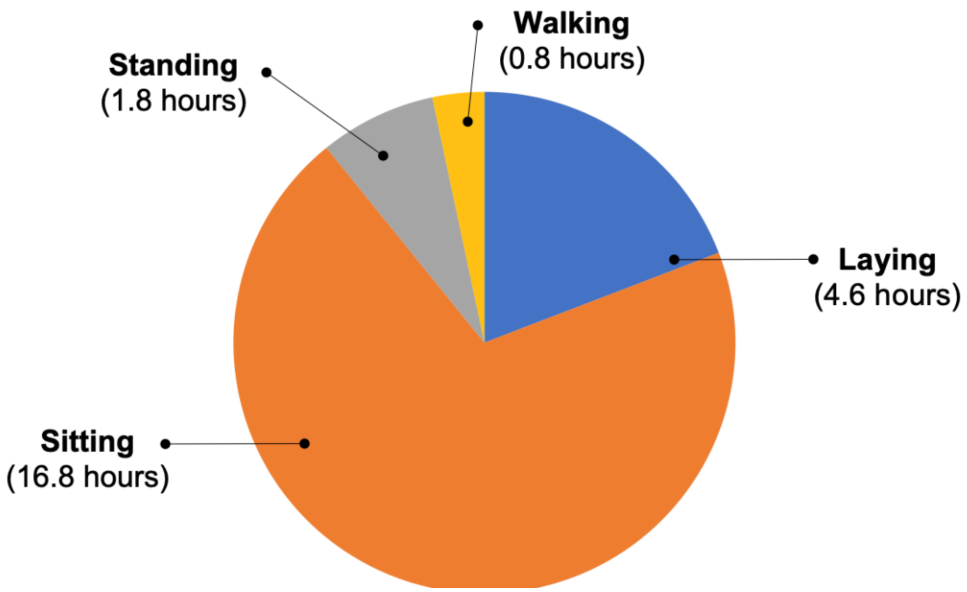

McMaster University HEALTH SCIENCES

Department of Medicine, Division of Geriatric Medicine
St. Peter's Hospital
158-88 Maplewood Avenue
Hamilton, ON L8M1W9

Dr. Isabel B. Rodrigues
(905) 521-2100 EXT 12464
rodrigibl@mcmaster.ca

	<insert date>	<insert date>	<insert date>
Early morning (12:00 am – 7:59 am)	• X hours laying<br>• Y hours reading<br>• Z hours browsing internet	• X hours laying<br>• Y hours browsing internet<br>• Z hours talking on phone	• X hours watching TV<br>• Y hours laying<br>• Z hours browsing internet
Late morning (8:00 am – 11:59 am)	• X hours browsing internet<br>• Y eating<br>• Z hours watching TV	• X hours browsing internet<br>• Y eating<br>• Z hours watching TV	• W hours browsing internet<br>• X hours watching TV<br>• Y hours napping<br>• Z hours eating
Afternoon (12:00 pm – 4:59 pm)	• X hours driving<br>• X hours browsing internet	• X hours watching TV<br>• Y hours napping<br>• Z hours eating	• X hours watching TV<br>• Y hours browsing internet
Evening (5:00 pm – 8:59 pm)	• W hours food preparation (sitting)<br>• X hours eating<br>• Y hours cleaning-up (sitting)<br>• Z hours watching TV	• X hours food preparation (sitting)<br>• Y hours eating<br>• Z hours watching TV	• X hours eating<br>• Y hours watching TV
Night (9:00 pm – 11:59 pm)	• X hours watching TV<br>• Y hours laying	• X hours watching TV,	• X hours watching TV

If you have any questions, please do not hesitate to call or email me. We appreciate your participation and hope that this has been a positive experience for you.

Sincerely,

Dr. Isabel B. Rodrigues
